# Stress exposure status and associated factors among Chinese People's Armed Police personnel: A cross-sectional study

**DOI:** 10.3389/fpsyt.2022.1000981

**Published:** 2022-11-03

**Authors:** Nan Li, Yongzhong Zhang, Shike Hou

**Affiliations:** ^1^College of Management and Economics, Tianjin University, Tianjin, China; ^2^Institute of Disaster and Emergency Medicine, Tianjin University, Tianjin, China

**Keywords:** PAP personnel, stress exposure status, mental health, mass gathering deployment, health service utilization

## Abstract

**Background:**

MG (Mass gathering) deployment is one of the primary duties of Chinese People's Armed Police (PAP) personnel. Due to prolonged and repeated deployments in difficult conditions and harsh climates, military personnel are exposed to multiple stressors.

**Objectives:**

This study aims to understand the stress exposure status of armed police personnel during MG deployment and to explore its influencing factors.

**Methods:**

A cross-sectional study was conducted among PAP in 2021. We used a cluster random sampling to select 960 PAP personnel. Binary logistic regression was used to examine whether the stress exposure status was associated with factors such as demographics, health service utilization, and MG deployment.

**Results:**

Among 960 PAP personnel,83% of PAP personnel participated in MG in the past month, and 23.1% of PAP personnel suffered stress. The chi-square test showed that there were significant differences in MG'cycle time (*p* < 0.05). The binary logistic regression results showed that satisfaction with medical skills (*p* = 0.008), satisfaction with health environment (*p* = 0.031), satisfaction with medicine (OR = 0.640, 95%CI:0.436,0.938), and seeking health services (OR = 5.36, 95%CI:2.316,12.402) were associated with stress exposure status. However, age, and length of military service did not have any association with the stress exposure status of PAP personnel in this study.

**Conclusion:**

This study demonstrated that stress exposure status among PAP personnel was associated with MG deployment, and health service utilization. These findings can help policy-makers and clinicians to relieve the stress of the armed police personnel, as well as provide a basis for developing military health service security plans.

## Introduction

Military service has been associated globally with an elevated risk of negative mental health outcomes including posttraumatic stress disorder (PTSD), depression, substance use, and suicidal behavior (1–5). In China, the detection rate of psychological problems in military personnel is much higher than that in the general population, and the level of psychological health is lower than the domestic adult norm (6–9). The maintenance of good mental health for all military personnel is one of the main goals of military medicine.

Due to the unique nature of PAP personnel's work, military personnel are burdened not only with the heavy intellectual, theoretical and training stress of modern military innovation, but also with the responsibilities of their country and their people, as well as with social and moral pressures. In particular, in order to maintain the safety of personnel, PAP personnel ensure the smooth running of mass gatherings, and improve military proficiency, often take part in different types of MG deployments, including large-scale sporting events (Olympic Games), large-scale political events, large-scale military training, large-scale festivals, etc. Therefore, they often have many experiences that are different from those of civilians (10).

These deployments where military personnel may be exposed to a number of hazards that affect health functioning. The adverse effect of prolonged and repetitive deployment of troops this highly stressful environment leads to many combat stress behaviors as well as misconduct behaviors (11). It not only brings physical injuries leading to loss of physical function but also exposes military personnel to extreme stress, such as harsh environmental conditions and combat (12–14). This can lead to various mental health problems such as depression, compulsion, interpersonal sensitivity, anxiety and other aspects of illness (15, 16). Furthermore, physical ailments and mental health problems negatively impact health, potentially resulting in sleep problems, appetite changes, digestive problems, pain, and fatigue (17).

Although stressful deployment experiences such as Participating MG have been associated with increased negative mental health outcomes in military populations, Participating MG experiences are not the only factor that contributes to stress among PAP personnel. American and Israeli studies have isolated various risk factors that influence the incidence of injury and excessive stress symptoms (I&ESS) during basic military training (BMT) (18, 19). In addition to other risk factors, such as being female, underweight or overweight (20, 21), active tobacco smoking (22), being of a certain age (23) or belonging to a specific ethnic group (24), physical fitness at the beginning of the training period has been found to be a key predictor for a higher incidence of I&ESS during BMT (25). During the Beijing Olympics, a researcher found that the main factors affecting the mental health of military personnel include poor psychological defenses, stressors, personality introversion, family education method, lack of social support, passive ways of coping with stress, previous and family history and marital status (26).

Most studies in this field deal with the influences that contribute to PTSD in veterans, but little is known about the research that affects the stress profile of PAP personnel during MG deployment. By aeessing all of the current literature, the aim of this study was to (1) examine sociodemographic factors, participating in MG in the past month, and health service utilization-related factors that are associated with the stress exposure status of PAP personnel on active duty and (2) provide reference data that can help in monitoring and maintaining the mental health of PAP personnel.

## Methods

### Study population

We conducted a cross-sectional survey using a paper questionnaire in China from June, 2021 to December, 2021, which was completed in collaboration with the PAP. The study was based on the method of cluster random sampling, we randomly selected six primary army units of the PAP that had mass gathering deployment tasks, and recruited 1,133 participants. The criteria for inclusion were: (1) PAP personnel who volunteered to participate in the survey; (2) PAP personnel who have not received mental health services in the past month; (3) PAP personnel who will be going to mass gathering deployment. The criteria for exclusion were: Assistant nurse of PAP.

The study was based on the method of cluster random sampling, we randomly selected six primary army units of the PAP that had mass gathering deployment tasks, and recruited 1,133 participants. The criteria for inclusion were: (1) PAP personnel who volunteered to participate in the survey; (2) PAP personnel who have not received mental health services in the past month; (3) PAP personnel who will be going to mass gathering deployment. The criteria for exclusion were: Assistant nurse of PAP.

A nationwide sample of 1133 PAP personnel with mean age of 20 years were drawn. Of 1133 targeted individuals, we excluded 173 PAP personnel who provided incomplete information. Hence, the study sample included 960 PAP personnel aged 18–42 years (958 male, 99.79% of the overall sample). The female PAP personnel only comprised 0.3% of the survey population, therefore, gender should not be a factor in the chi-square test and logistic regression analysis. The response rate was 960/1,133 = 85%.

The questionnaire was completed voluntarily by each participant according to the wishes of the PAP personnel. All participants were able to submit, terminate and revise the questionnaire directly, even if they started completing it with prior consent. Ethical approval was obtained from Tianjin University Ethics Committee (TJUE—approved 17, June 2022).

### Questionnaire

Based on previous literature, authors developed a semistructured questionnaire to collect data. We performed the KMO test and Bartlett sphericity test, the result indicated suitable values: KMO = 0.724 (27). In addition, it was reviewed by two experts (one professor of military medicine, and another expert of mass gathering medicine). The questionnaire comprised 35 items and four sections, including personal profile, environment of participating in MG deployment in the past month, health service utilization, stress exposure status.

Personal information: such as age, gender, education, length of military service, rank and identity level were obtained through a questionnaire.

Information about MG deployment: participated in MG deployment in the past month (yes vs. no), frequency of MG deployment (1–2 times/year, 3–5 times/year, 5 times and above/year), types of MG deployment (political meetings, large celebrations, military training activities, sports events, economic meetings, others).

Information about stress: stress exposure status (yes vs. no), sources of stress (family factor, working capability, superior pressure, interpersonal relationship, other factors). Note that in this study, we used self-reports from PAP personnel to understand their stress exposure status.

Information about health services utilization status: satisfaction with medical skills (very satisfied, relatively satisfied, moderate, somewhat dissatisfied, very dissatisfied), satisfaction with health environment (very satisfied, relatively satisfied, moderate, somewhat dissatisfied, very dissatisfied), whether medicines met demand (yes vs. no), demand for health services (open-ended question).

### Statistical analysis

First, the sociodemographic characteristics of PAP personnel were analyzed with descriptive statistics and are presented in percentages. To eliminate the effect of confounding factors on mental health status, we used a chi-square test preliminarily analyze various independent variables. Binary logistic regression analysis used demographic variables, MG deployment variables, and health service- related variables as independent variables, and the stress exposure status of self- reported as binary dependent variables were conducted to identify factors associated with stress status.

All statistical analyses were performed by the Statistical Package for the Social Sciences (SPSS, version 22.0). *P* < 0.05 was considered statistically significant. Hosmer–Lemeshow goodness-of-fit was used to test the model's fitness. The odds ratio (OR) with a 95% confidence interval was used to identify factors associated with stress status among PAP personnel.

## Results

### Sociodemographic characteristics of the study participants

To examine demographic characteristics, we collected data on gender, age, education level, stress exposure status, and participated in MG in past month (yes or not) (Table 1). The present study included 960 participants. Among all participants, the majority of respondents were male (99.7%). The age range was from 18 to 42 years, and the average age was 22.5. The most common age group was 17–24 years, comprising 82.6% of the study sample, and 785 (81.8%) PAP personnel have less than 5 years of military service. The percentage of subjects who were college graduates or higher was 55.4%. In addition, 83% of PAP personnel participated in MG in past month, and only 33(3%) received health service.

**Table 1:** Sociodemographic characteristics of PAP personnel.

**Observations**	**Stress exposure status**		**Total**	**χ^2^/F**	***p***
	**Yes**	**No**	**N (%)**		
**Gender**				-	-
Male	221 (99.5)	737 (99.9)	958 (99.7)		
Female	1 (0.5)	1 (0.1)	2 (0.3)		
**Age**				1.971	0.373
17–24	178 (80.2)	615 (83.3)	793 (82.6)		
25–34	44 (19.8)	121 (16.4)	165 (17.2)		
35–44	0	2 (0.3)	2 (0.2%)		
≥45	0	0	0		
**Length of military service (year)**				3.743	0.291
< =5	159 (71.6)	561 (76)	785 (81.8)		
5–10	48 (21.6)	141 (19.1)	141 (14.7)		
11–15	12 (5.4)	33 (4.5)	29 (3)		
16–20	3 (1.4)	3 (0.4)	6 (0.6)		
**Education**				22.225	0.001^***^
Primary school	1 (0.5)	2 (0.3)	3 (0.3)		
Junior high school	3 (1.4)	5 (0.7)	8 (0.8)		
Secondary school	32 (14.4)	125 (17.5)	161 (16.8)		
Senior high school	60 (27)	196 (26.6)	256 (26.7)		
College	86 (38.7)	347 (47.0)	433 (45.1)		
Bachelor	40 (18)	58 (7.9)	98 (10.2)		
Master and above	0 (0)	1 (0.1)	1 (0.1)		
**Rank**				13.709	0.018^*^
Recruits	5 (2.3)	37 (5)	42 (4.4)		
Conscripts	71 (32)	236 (32)	307 (32.0)		
Non-commissioned officers	133 (59.9)	447 (60.6)	580 (60.4)		
Fellow	2 (0.9)	2 (0.3)	4 (0.4)		
Cadres below battalion	11 (5)	12 (1.6)16 (2.3)	23 (2.4)		
Cadres above regiment	0	4 (0.5)	4 (0.4)		
**Participated in MG in the past month**				4.415	0.036^*^
Yes	174 (78.4)	623 (84.4)	797 (83)		
No	48 (21.6)	115 (15.6)	163 (17)		

* *p* < 0.01;

*** *p* < 0.001.

To simplify the model and make the fit better, we used a chi-square test to select independent variables that had significant differences before the binary logistic analysis was performed. The chi-square test showed that there were significant differences in stress exposure status and education (χ^2^ = 22.225, *p* = 0.001), rank (χ^2^ = 13.709, *p* = 0.018), and participating in MG in the past month (χ^2^ = 4.415, *p* = 0.036). Whereas, stress exposure status is not associated with age (χ^2^ = 1.971, *p* = 0.373), gender (χ^2^ = 0.814, *p* = 0.367), length of military service (χ^2^ = 3.743, *p* = 0.291).

### MG-related factors

Our results showed that 222 (23.1%) of PAP personnel suffered from stress, and 738 (76.9%) expressed that they were in good mental health and had not suffered from stress. Regarding related factors about MG deployment, 369 (38.4%) participated in sports meetings, 679 (70.9%) were outdoor, 655 (68.2%) of PAP personnel considered the weather during MG deployment was hot, 37.4% in high popular density,644(68.4%) participated in 1–2 times of MG, 363 (39.5%) participated in an MG' cycle time is 1 week.

This study used multiple response analysis combined with chi-square tests to analyze the relationship between stress exposure status and MG deployment. The results in Table 2 show the type of MG (χ^2^ = 16.436, *p* = 0.006), MG frequency (χ^2^ = 8.141, *p* = 0.043), MG cycle time (χ^2^ = 17.815, *p* = 0.000), MGs climate (χ^2^ = 16.862, *p* = 0.002) have significant impacts on stress exposure status of PAP personnel. While MG position (χ^2^ = 4.346, *p* = 0.361), MG environment (χ^2^ = 2.744, *p* = 0.433) were irrelevant to the stress exposure status of PAP personnel.

**Table 2:** MG deployment factors associated with PAP personnel's stress exposure status.

**Observations**	**Stress exposure Status**		**Total**	**χ^2^/F**	***p*** **-value**
	**Yes**	**No**	**N (%)**		
	222 (23.1%)	738 (76.9%)	960		
**Type of MG**				16.436	0.006^**^
Large political meetings	56 (23.1)	186 (76.9)	242 (25.2)		
Large-scale celebrations	64 (21.3)	236 (78.7)	300 (31.3)		
Large training activities	57 (27.4)	151 (72.6)	208 (21.7)		
Sports meetings	81 (22.0)	288 (78.0)	369 (38.4)		
Large economic meetings	49 (35)	91 (65.0)	140 (14.6)		
Other	63 (31.2)	139 (68.8)	202 (21)		
**MG environment**				2.744	0.433
Indoor	98 (21.5)	358 (78.5)	456 (47.6)		
Outdoor	169 (24.9)	510 (75.1)	679 (70.9)		
High population density	90 (25.1)	268 (74.9)	358 (37.4)		
Low population density	54 (26.5)	150 (73.5)	204 (21.3)		
**MG climate**				16.862	0.002^**^
Hot	174 (26.6)	481 (73.4)	655 (68.2)		
Cold	38 (23.2)	126 (76.8)	164 (17)		
High humidity	29 (29)	71 (71)	100 (10.4)		
Dry	39 (20.1)	155 (79.9)	194 (20.2)		
Other	20 (12.6)	139 (87.4)	159 (16.5)		
**MG frequency**				8.141	0.043^*^
0	8 (19.5)	33 (80.5)	41 (4.4)		
1–2	137 (21.3)	507 (78.7)	644 (68.4)		
3–5	52 (25.9)	149 (74.1)	201 (21.3)		
>5	22 (36.1)	39 (63.9)	61 (6.5)		
**MG cycle time**				17.815	0.000^***^
1day	37 (21.1)	138 (78.9)	175 (19)		
1week	72 (19.8)	291 (80.2)	363 (39.5)		
2weeks	31 (18.5)	137 (81.5)	168 (18.3)		
>2weeks	86 (38.6)	178 (67.4)	264 (28.7)		
**MG position**				4.346	0.361
Sentry post	165 (24.8)	500 (75.2)	665 (69.3)		
Temporary duty stations	23 (22.8)	78 (77.2)	101 (10.5)		
On site	37 (21.3)	137 (78.7)	174 (18.1)		
Mobile patrol	39 (30.7)	88 (69.3)	127 (13.2)		
Standby for troop presence	58 (22.6)	199 (77.4)	257 (26.8)		

* *p* < 0.05;

** *p* < 0.01;

*** *p* < 0.001.

### Correlation between stress exposure status and possible risk factors

To eliminate the effect of confounding factors on stress exposure status, we included statistically significant factors from Table 1 in the binary logistic regression model. The Hosmer-Lemeshow test indicated equal goodness-of-fit (*p* = 0.442). The binary logistic regression result is shown in Table 3. The results showed that PAP personnel who keep relatively satisfied with medical skills (vs. somewhat dissatisfied, *p* = 0.006), keep moderate satisfied with medical skills (vs. somewhat dissatisfied, *p* = 0.01), keep very satisfied with health environment, (vs. somewhat dissatisfied, *p* = 0.007), keep relatively satisfied with health environment (vs. somewhat dissatisfied, *p* = 0.022), medicine supply can meet MG deployment's needs (vs. can't meet needs, *p* = 0.022), seek health services (vs. not seeking health service, *p* = 0.000) were significantly associated with stress exposure condition of the PAP personnel.

**Table 3:** Binary logistic regression analysis of PAP personnel's stress exposure status.

**Independent variables**	***P***	**OR**	**95%CI**	
			**Lower limit**	**Upper limit**
**Rank**	0.580			
Recruits	0.124	0.444	0.157	1.250
Conscripts	0.082	0.405	0.146	1.123
Non-commissioned officers	0.942	1.090	0.108	10.963
Fellow	0.339	0.502	0.122	2.061
Cadres below battalion	0.999	-	0.000	.
Cadres above regiment				
**Education**	0.056			
Primary school	0.701	1.765	0.097	32.011
Junior high school	0.290	3.892	0.314	48.243
Secondary school	0.328	3.495	0.285	42.876
College	0.230	4.629	0.379	56.473
Bachelor	0.647	1.811	0.142	23.099
Master and above	1.000	1.860	0.000	.
**Participated in MG in the past month**	0.075	0.686	0.453	1.039
**Seek health services**	0.000^***^	5.360	2.316	12.402
**Medical skills**	0.008^**^			
Very good	0.221	0.728	0.438	1.210
Relatively satisfied	0.006^**^	0.397	0.205	0.767
Moderate	0.010^*^	0.273	0.102	0.730
**Health environment**	0.031^*^			
Very satisfied	0.007^**^	0.505	0.308	0.827
Relatively satisfied	0.022^*^	0.404	0.186	0.876
Moderate	0.950	1.064	0.150	7.530
**Medicine supply**	0.022^*^	0.640	0.436	0.938
**Constant**	0.882	0.813	-	

CI, confidence interval.

* *p* < 0.05;

** *p* < 0.01;

*** *p* < 0.001.

The binary logistic regression demonstrated that among PAP personnel, seeking health services, satisfaction with medical skills, satisfaction with health environment, medicine supply were the most significant predictors. In particular, the group that keep relatively satisfied with medical skills (OR = 0.397,95%CI:0.205–0.767) and the group that keep very satisfied with environment (OR = 0.505,95%CI:0.308–0.827) showed lower odds of stress exposure status than other groups. the group that keep moderate satisfied with health environment (OR = 1.064,95%CI:0.150–7.530) and the group that seeking health services (OR = 5.360,95%CI:2.316,12.402) showed greater odds of stress exposure status than other groups.

## Discussion

This is the first study to examine Chinese PAP personnel's stress exposure status during MG deployment and to explore its influencing factors. This evaluation was essential to improve the mental health of PAP personnel. The results revealed that 738 (76.9%) expressed that they were in good mental health and had not suffered from stress, At the same time, 222 (23.1%) PAP personnel who suffered from stress. However, previous studies have found that 46.7 of the Army training officers and soldiers suffer from stress (28). Besides, a UK study surveying individuals deployed to Iran found that 26.3% of reservists may experience common mental health problems (29). It is obvious that the result of this study is lower than those of previous similar studies. The reasons for this outcome are complex. On the one hand, the selection criteria for PAP personnel are very strict to exclude those with poor mental health, as well as those with poor tolerance. Besides, severely maladjusted PAP personnel were separated from the military at an early stage and were not included in this study. The last but not least reason may be that these PAP personnel experienced an initial difficult adaptation period, gained relevant experience and made positive changes from the stress associated with the training (30).

We found that there were significant differences in stress exposure status in terms of education and rank. These findings are consistent with a study by Laurel (31). Compared to low-educated PAP personnel, high-educated PAP personnel have higher achievement motivation and self-demand, and suffer more stress. Previous studies have shown that low ranking officers and soldiers are at high risk for stress reactions (32). Some studies showed that with the increase of age and military age, can lead to an effect on sleep due to increased psychological stress of PAP personnel (33). We did not observe associations between age, length of military service and stress exposure, which is in contrast to previous studies. It is possible that in this study, PAP personnel may have attended mental health training courses, maintained a youthful and optimistic mindset, and planned for their future careers. As a result they suffered less anxiety and stress.

Mass gatherings security is an important part of military occupations and cannot be avoided. Participation in numerous military maneuvers and living in isolation for long periods of time can cause psychological stress in PAP personnel (34). The results of our study indicate that the stress exposure status of PAP personnel is different whether they participate in mass gatherings deployment, which is consistent with a US study (35). In addition, Our study is in line with previous study, different climates, different MG types and different frequencies have significant impacts on stress exposure status of PAP personnel. Furthermore, MG' cycle time also affected stress exposure status, PAP personnel may experience some circadian dysrhythmia (e.g., jet lag, which interferes with the sleep cycle) as well as mild environmental disorientation, often due to a change in geographical location. This may lead to debilitating medical and mental health issues that require treatment (36, 37). As the length of deployment increases, the risk of adverse mental health effects increases (38–42). Furthermore, first-time deployers may be considerably more vulnerable (43). During intense MG deployments, the adverse effects of prolonged and repeated deployments of troops in these highly stressful, difficult conditions and harsh climates lead to many incidents of misconduct (11). Therefore, it is necessary for military policy-makers to rationally plan the schedule and frequency of MG deployment and improve the conditions for MG deployment.

With China's rapid economic development in recent years, the living conditions of PAP personnel have also improved dramatically, which has had an obvious beneficial effect on their mental health. However, the utilization of health services for PAP personnel is also an issue worthy of attention. Our study also examined some risk factors of health service utilization associated with stress exposure in PAP personnel. For example, medical skills, health environment, medicine supply were significantly associated with stress exposure status of the PAP personnel. In China, a multi-directional coordination mechanism had been established between military hospitals and basic military units. This improved the efficiency of medical examination, assessment, treatment, health care and rehabilitation. When PAP personnel seek medical treatment, doctors can easily access relevant medical examination outcomes and health monitoring information to make treatment more targeted (44). With the development of the Internet, the unified integration of clinical departments and specialist teams within hospitals can provide remote pathology diagnosis, imaging diagnosis, specialist consultation, surgical guidance and other medical services to primary medical organizations in remote and underdeveloped areas. This has formed an innovative approach to the whole medical service based on general practitioners, clinical experts as the core, network platform as the basis, grass-roots points as the extension, and guidance for patients to rationalize medical care as the main service content. However, there are still very few people who have a bad seeking medical service experience due to poor doctors' skills, poor health environment and inadequate medicine supply, which can cause anxiety and stress for PAP personnel, especially those who have just returned from mass gathering deployment. There are a few potential explanations to these outcomes. First, due to geography, resources such as medical staff, medical equipment and drugs are unevenly distributed. In addition, decline in medical skills due to loss of medical and nursing talent. Finally, the complicated transfer approval process has resulted in PAP personnel not receiving timely medical care.

There is a general shortage of medical personnel in the grassroots medical system, because the PAP personnel participated in many mass gathering deployments and receive training in the field for a long time. In addition, in primary medical organization, there are aging medical facilities and equipment, incomplete medicine type, and inadequate medicine supply, resulting in PAP personnel not receiving timely medical service and even needing to be transferred to military hospitals. The OR indicates that the group who keep relatively satisfied with medical skills and the group who keep very satisfied with health environment had significantly lower odds of having no stress exposure. Therefore, from the perspective of health service utilization, meeting the health service needs of PAP personnel and improving their satisfaction with health care are also important means of reducing stress exposure in PAP personnel.

It is critical to eliminate the effect of stress to improve the PAP personnel' performance of diverse military tasks. Through training in stress resistance, an important way to enhance the psychological resilience of officers and soldiers. The training enables officers and soldiers to acquire strategies for coping with stress and to gain inner experience and practical experience. Besides, psychologists use the military integrated information network to develop psychological services platform to provide timely psychological counseling services to PAP personnel who have completed mass gathering deployment (45). PAP personnel can use the psychological service platform to conduct a mental health test and learn psychologically relevant knowledge in a targeted manner based on the test results (46). In addition, MBSR (Mindfulness-based stress reduction) could be a promising health intervention for improving mental health and reducing stress in Chinese military. It can reduce anxiety and depression, significantly improve sleep quality, and promote physical and mental health (47).

## Limitations and future directions

The main limitation of this study is that we lack data on a number of important factors (e.g., scale of MG, the physical health of PAP personnel) that have been shown to be associated with mental health in previous studies (48, 49). Another limitation is that our study, which used self-reported measures of stress exposure may have limited reliability. Future studies should use standardized measurement scales (e.g., SCL-90, PSTR) or objective military records to report on stress exposure. Nevertheless, the implications of our findings are significant. First, we are an innovative and scarce study addressing the stress exposure status of Chinese PAP personnel during large event deployments. Second, these findings may help to improve the mental health status of Chinese PAP personnel and contribute to the diversification of the Chinese military medical department. Finally, future research will analyze and monitor the stressors of PAP personnel to determine how to improve interventions for the mental health of PAP personnel.

## Conclusion

This study provides the first evidence of demographic, health service utilization and MG deployment-related risk factors for military stress exposure, though most derive from cross-sectional studies. Our study found that 23.1% of PAP personnel in China suffer stress during MG deployment. MG service time, MG climate, and satisfaction with health environment. are the most important predictors of stress exposure status. Understanding the risks and consequences of MG deployment-related mental health problems among active-duty PAP personnel allows for better prevention and treatment. This study will help policy-makers and clinicians better care for PAP personnel with stress problems, and provide a basis for developing health service security plans.

## Data availability statement

The raw data supporting the conclusions of this article will be made available by the authors, without undue reservation.

## Ethics statement

The studies involving human participants were reviewed and approved by Tianjin University Ethics Committee, Tianjin University. The patients/participants provided their written informed consent to participate in this study. Written informed consent was obtained from the individual(s) for the publication of any potentially identifiable images or data included in this article.

## Author contributions

SH and YZ co-developed and co-led the study. NL was responsible for collecting data and writing the first draft. YZ and NL conducted the analysis and wrote up the results. NL, SH, and YZ critically reviewed and revised the manuscript. All authors read and approved the final manuscript.

## Funding

This research was funded by the National Key Research and Development Program of China (Grant No. 2021YFC2600504) and Key Program of National Natural Science Foundation of China (Grant No. 71533008).

## Conflict of interest

The authors declare that the research was conducted in the absence of any commercial or financial relationships that could be construed as a potential conflict of interest.

## Publisher's note

All claims expressed in this article are solely those of the authors and do not necessarily represent those of their affiliated organizations, or those of the publisher, the editors and the reviewers. Any product that may be evaluated in this article, or claim that may be made by its manufacturer, is not guaranteed or endorsed by the publisher.
